# Aqueous Humor Mediator and Cytokine Aberrations in Diabetic Retinopathy and Diabetic Macular Edema: A Systematic Review and Meta-Analysis

**DOI:** 10.1155/2019/6928524

**Published:** 2019-11-23

**Authors:** Jingyang Wu, Yifan Zhong, Song Yue, Kaibo Yang, Guisen Zhang, Lei Chen, Lei Liu

**Affiliations:** ^1^Department of Ophthalmology, The First Hospital of China Medical University, Shenyang 110001, China; ^2^Hohhot Chao Ju Eye Hospital, Hohhot 010000, China; ^3^Department of Public Service, The First Hospital of China Medical University, Shenyang 110001, China

## Abstract

**Purpose:**

To evaluate the relationship between the aqueous humor levels of VEGF, TNF-*α*, IL-10, IL-6, IL-12, MCP-1, and IP-10 with DR/DME.

**Methods:**

PubMed, Web of Science, Embase, China National Knowledge Infrastructure (CNKI), and Wanfang databases were searched up to October 2018. Systematic review and meta-analysis were conducted.

**Results:**

18 studies comprising 362 cases with DR (100 with DME) and 620 controls without DR were included in this meta-analysis. There was a significant association between VEGF levels in the aqueous humor and DR (standardized mean difference (SMD) 1.94 (95% CI 1.05-2.83)) and DME (1.07 (0.71, 1.42)). Furthermore, a significant correlation was observed between levels of IL-6 and DR (3.53 (0.37, 6.69)), and similarly correlation with DME (1.26 (0.30, 2.21)). The relationship between MCP-1 and DR and DME was significant, in which the SMD was (0.49 (0.09, 0.89)) and (1.49 (0.78, 2.20)), respectively. However, IL-12, IP-10, and TNF-*α* had no correlation with DR and DME, whereas there was a significant relationship between IL-8 and DME (1.68 (0.97, 2.40)).

**Conclusion:**

Elevated levels of VEGF, IL-6, and MCP-1 in the aqueous humor were associated with the risk for the presence of DR, and levels of VEGF, IL-6, IL-8, and MCP-1 were associated with the risk of DME. Furthermore, these biomarkers may be used as potential predictors or therapeutic targets for DR/DME.

## 1. Introduction

DR is one of the most common microcomplications of diabetes and has become the major cause of decreased vision and blindness in adults aged 20-74 years [1]. Recently, the disturbance of inflammatory reaction may play an important role in the numerous researches of the complex pathogenesis of DR [2]. Previous studies have shown that deregulation of immune responses associated with diabetes can induce high expression of various mediators resulting in the development of DR [3]. Furthermore, analysis of intraocular humors obtained from DR patients has indicated that some of the mediators (cytokines or chemokines) may be responsible for the pathogenesis of DR. The immune and inflammatory factors, e.g., VEGF, TNF-*α*, IL-6, IL-8, and MCP-1, have been observed elevated in both aqueous humor and vitreous fluids in patients with DR [4, 5]. Hence, the altered concentrations of various cytokines regarding these mechanisms may serve as important biomarkers to assess early detection or treatment of DR.

Diabetic macular edema (DME) is also one of the most serious causes of visual disability and blindness and affects approximately 14% of patients with diabetes [6]. The pathophysiology of DME is multifactorial, complicated, and partly still unknown. Several mediators, including VEGF, as well as other inflammatory cytokines, such as IL-6, IL-8, TNF-*α*, IP-10, and MCP-1, are among the possible responsible mechanisms or are involved in the development of DME [7–9]. However, the exact cytokines in the aqueous humor regarding the pathogenesis of DME remain unknown.

Taken together, the association between levels of inflammatory cytokines in the aqueous humor and DR is still controversial [10, 11]. Moreover, there have been no systematic review and meta-analysis to evaluate the available evidence on the association of the aqueous humor levels of inflammatory cytokines with the risk of DR or DME. Herein, we perform this systematic review and meta-analysis to investigate the relationships between these biomolecule levels in the aqueous humor and the risk of DR and DME.

## 2. Materials and Methods

### 2.1. The Study Identification and Search Strategy

We identified the relevant evidences of mediators or cytokines in the aqueous humor among patients with type 2 diabetes mellitus (T2DM) by systematically searching PubMed, Embase, Web of Science, China National Knowledge Infrastructure (CNKI), and Wanfang databases, from inception to October 2018. The following key words were used to perform searches in the databases mentioned above: (inflammation, inflammatory markers, inflammatory biomarkers, inflammatory mediators, inflammatory cytokines) or (vascular endothelial growth factor, VEGF, tumor necrosis factor, TNF-*α*, interleukin 6, IL-6, interleukin 8, IL-8, interleukin 10, IL-10, monocyte chemoattractant protein-1, MCP-1), and (diabetic retinopathy, retinopathy, DR) or (diabetic macular oedema, diabetic macular edema), and (aqueous humor, aqueous humour), without year restriction. Moreover, an extensive hand search was performed and the additional relevant studies were further reviewed in reference lists. The selection process was according to the Preferred Reporting Items for Systematic Reviews and Meta-Analyses (PRISMA) flow diagram [12].

### 2.2. Inclusion and Exclusion Criteria

The inclusion criteria were as follows: (1) the study should report the correlation between the aqueous humor levels of mediators or cytokines (VEGF, TNF-*α*, IL-6, IL-8, IL-12, IP-10, and MCP-1) and DR/DME; (2) it should be written in English or Chinese with the full text available; (3) patients with any age, gender, region or race were considered.

Exclusion criteria were predefined as follows: (1) literature reviews, case reports, and cell lines or animal studies; (2) duplication: same studies came from different databases; (3) no sufficient data to perform meta-analysis; (4) no DM group or no health control group.

### 2.3. Data Extraction

Two reviewers (Lei Liu and Jingyang Wu) independently reviewed each included study. The disagreements on eligibility during the reviewing were discussed and resolved by the third reviewer (Song Yue). The following data were extracted by three reviewers (Yifan Zhong, Kaibo Yang, and Song Yue): the first author, location, year of publication, number of subjects in DR, DME, and control groups, definition of DR, cytokine measurements, and name of cytokines in detail. We defined that diabetic patients without retinopathy and/or matched healthy persons constituted the controls and patients with DR or DME were the cases.

### 2.4. Assessment of Methodology Quality

The quality of the included studies was assessed using the Newcastle-Ottawa scale (NOS) [13]. A quality score of more than or equal to seven on the nine-point NOS was considered to be high quality for included studies.

### 2.5. Statistical Analysis

The weighted standardized mean difference (SMD) was measured with 95% confidence interval (CI) using random-effects method. The subgroup analyses were conducted based on location, DR definition, and cytokine measurements. *I*^2^ tests were used to evaluate the statistical heterogeneity among the included studies, and the heterogeneity was considered statistical when *P* < 0.05 and *I*^2^ ≥ 50%. Additionally, sensitive analysis was also performed to evaluate the influences of individual studies on the final effect. The funnel plots were performed to evaluate the potential publication bias. This meta-analysis was performed using the Stata 11.0 statistical software (Stata Corp., College Station, TX). A two-tailed *P* less than 0.05 was considered as significant difference.

## 3. Results

### 3.1. Literature Search

**Figure 1:**
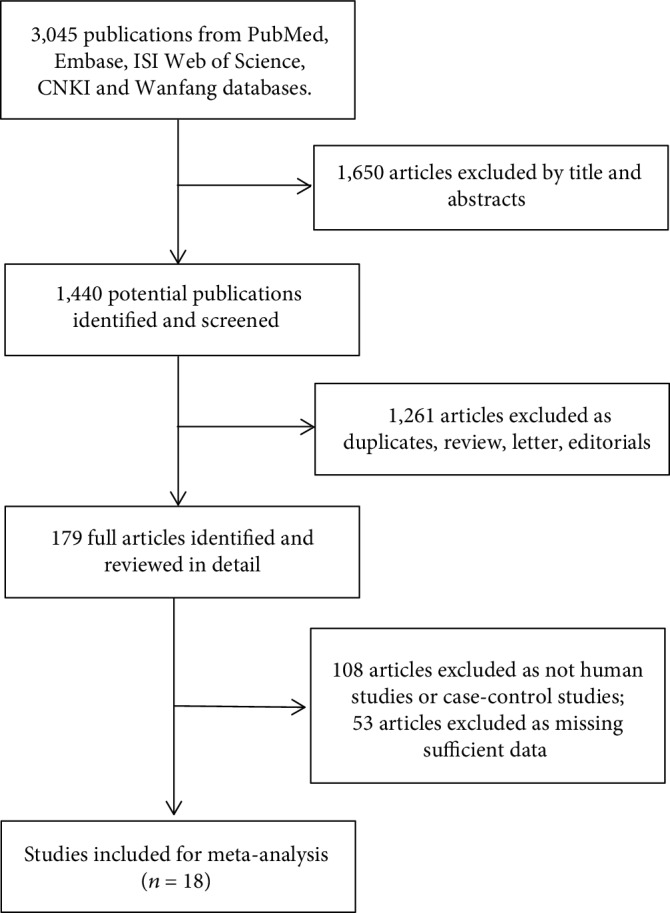
Flow diagram of study selection for the meta-analysis.

The study selection flowchart was shown in Figure 1. A total of 3,045 articles were identified from the databases (PubMed, Embase, Web of Science, CNKI, and Wanfang databases). 1,650 articles were excluded based on a review of the titles and abstracts, and 1,261 articles were excluded as duplicates, review, letter, and editorials. In addition, 108 articles were excluded as not human studies or case-control studies, and 53 articles were excluded as missing sufficient data. Finally, a total of 18 articles [7, 10, 11, 14–28] were included in this meta-analysis.

### 3.2. Characteristics of the Studies

18 case-control studies involving 362 cases with DR including 100 cases with DME and 620 controls with type 2 diabetes but without DR were included in the current meta-analysis. The characteristics of the included studies were presented in Table 1 and [Supplementary-material supplementary-material-1]. All cited references measured cytokines in different ways (e.g., Luminex xMAP suspension array, ELISA, and multiplex bead immunoassay). As shown in Table 2, according to the NOS checklist, 10 studies with scores ≥ 7 stars were considered high quality, and the remaining eight studies were medium quality for 6 stars.

### 3.3. Meta-Analysis and Subgroup and Sensitivity Analysis

#### 3.3.1. Analysis of VEGF

**Figure 2:**
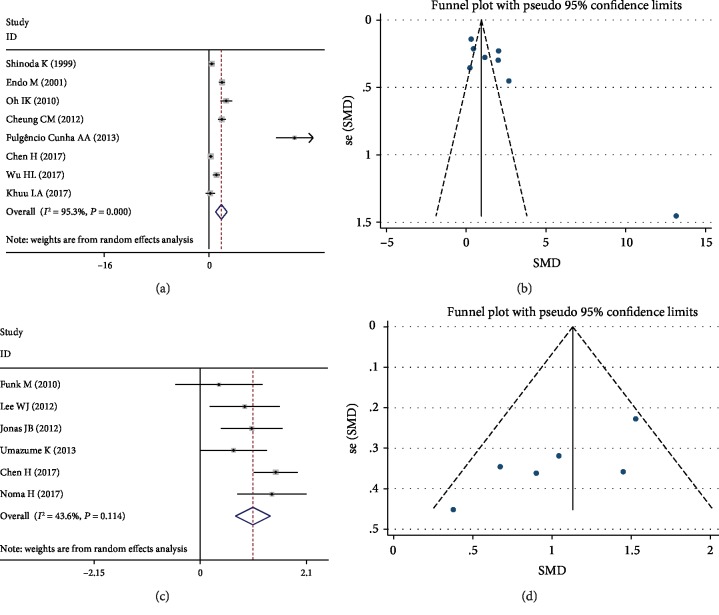


Figure 2(a) shows the pooled SMD derived from all 8 studies with the levels of VEGF in the aqueous humor of subjects with and without DR. The results present that there was a significant difference (SMD 1.94, 95% CI (1.05, 2.83)) but with heterogeneity in the aqueous humor level of VEGF between the DR and control groups. Further, the publication bias was not significant (Figure 2(b)).

**Figure 3:**
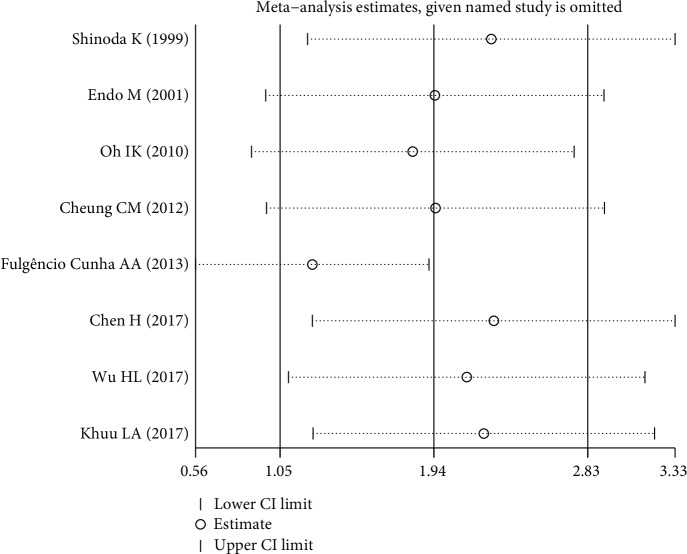


Bending with significant heterogeneity, it was necessary to perform subgroup and sensitivity analyses. Location, DR definition, and cytokine measurements may be the main causes of heterogeneity, but heterogeneity was still significant (*I*^2^ = 93.4%, 93.1%, and 97.8%, respectively) after the subgroup analysis (Table 3). A sensitivity analysis was used to evaluate the stability and reliability of the results (Figure 3). After removing the studies [11, 14, 21] that were contributing the most to the heterogeneity, the results did not change substantially (SMD 1.61, 95% CI (0.89, 2.33), *P* < 0.001, *I*^2^ = 85.8%).

There were significant differences in the aqueous humor level of VEGF between the DME and controls with SMD 1.07 (95% CI (0.71, 1.42), *P* < 0.001, Figures 2(c) and 2(d)) and no significant heterogeneity (*I*^2^ = 43.6%, *P* = 0.114). Moreover, the funnel plot showed that publication bias was also not significant.

#### 3.3.2. Analysis of IL-6

**Figure 4:**
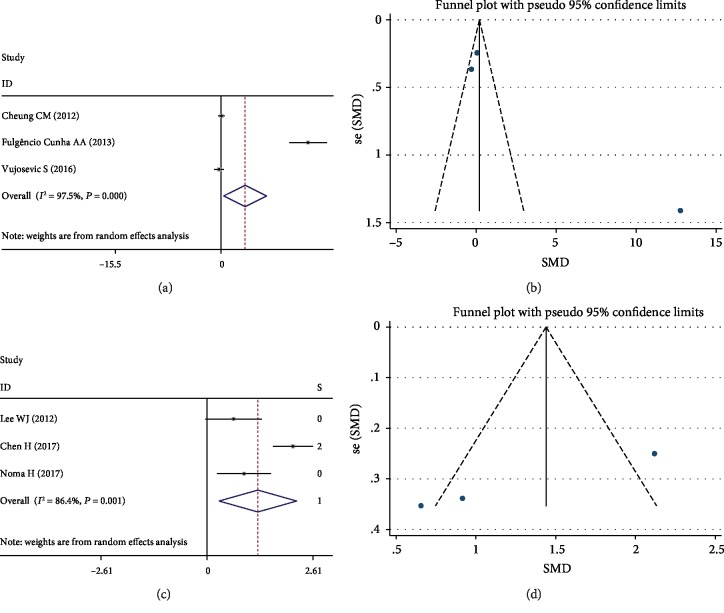


There were 3 studies that reported data on the relationship of IL-6 levels and DR among participants with T2DM. The results present that there were significant differences between DR and control groups (SMD 3.53, 95% CI (0.37, 6.69), *P* = 0.028), but with significant heterogeneity (Figure 4(a)). However, the publication bias was not significant (Figure 4(b)).

The results of IL-6 levels between the DME and control groups were shown in Figures 4(c) and 4(d). The pooled SMD was 1.26, and the 95% CI was 0.30 to 2.21 (*P* = 0.010, Figure 4(c)). The results present that there were significant differences but heterogeneity (*I*^2^ = 86.4%, *P* = 0.001) between these two groups. Subgroup analysis showed that cytokine measurements were the reason for obvious heterogeneity (*I*^2^ = 0.0%, *P* = 0.595). Performing subgroup analysis according to the cytokine measurements, the association was still significant (SMD 0.79, 95% CI (0.31, 1.27), *P* = 0.001). The funnel plot showed that publication bias was not significant (Figure 4(d)).

#### 3.3.3. Analysis of MCP-1

**Figure 5:**
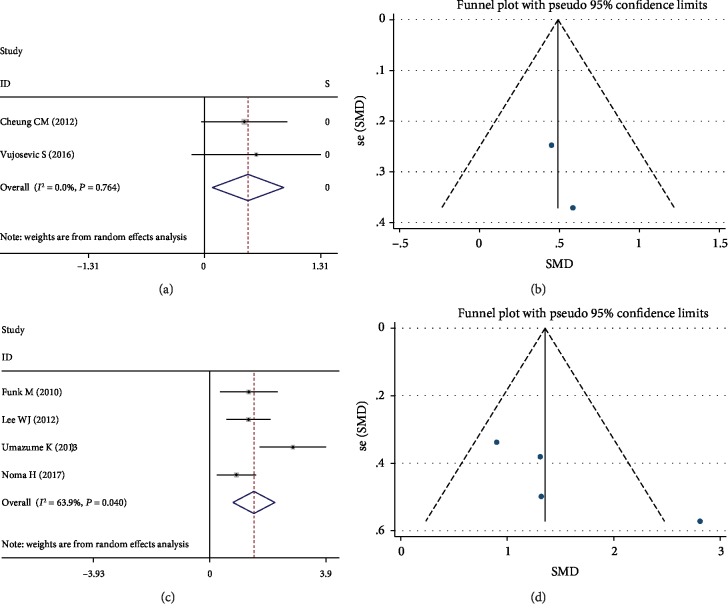


Compared to the controls, the DR patients resulted in significantly increased MCP-1 levels (SMD 0.49; 95% CI (0.09-0.89), *P* = 0.017) in aqueous humor (Figure 5(a)) with no significant heterogeneity between studies (*I*^2^ = 0.0%, *P* = 0.764). The publication bias was not significant (Figure 5(b)).

There were 4 studies that were included in the meta-analysis for the association of MCP-1 levels with the risk of DME. The pooled SMD for DME was 1.49 (95% CI (0.78-2.20), *P* < 0.001, Figure 5(c)) while with substantial heterogeneity among studies (*I*^2^ = 63.9%, *P* = 0.040). Subgroup analysis was performed according to the cytokine measurements (SMD 1.13, 95% CI (0.69, 1.57), *P* < 0.001), and the heterogeneity was not significant (*I*^2^ = 0.0%, *P* = 0.660). The funnel plot showed that publication bias was not significant (Figure 5(d)).

#### 3.3.4. Analysis of IL-8

The pooled SMD was 0.38 (95% CI (-0.05-0.81), *P* = 0.087) for the association between IL-8 levels in the aqueous humor and risk for DR (Table 4). Moreover, the funnel plot showed that publication bias was not significant.

**Figure 6:**
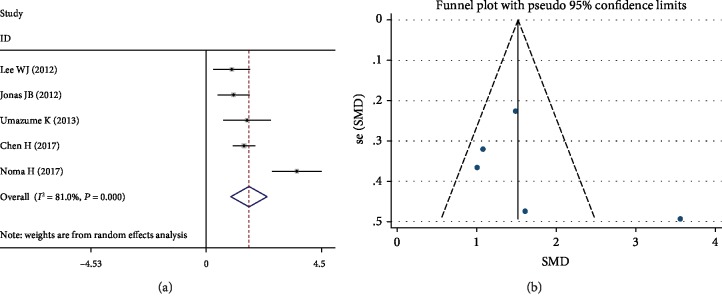


On the basis of the five studies, the overall SMD of DME was 1.68 (95% CI (0.97-2.40), *P* < 0.001) in IL-8 levels (Figure 6(a)). However, there was a significant heterogeneity among the five studies (*I*^2^ = 81.0%, *P* < 0.001). Dividing into subgroups according to countries, definition of DR, and cytokine measurements, respectively, heterogeneity was still evident (Table 4). After removing the study by Noma et al. [17], which was contributing to the significant heterogeneity, the heterogeneity was not significant (*I*^2^ = 0.0%, *P* = 0.525). The funnel plot showed that publication bias was not significant (Figure 6(b)).

#### 3.3.5. Analysis of IL-12

Three original studies were included in the current meta-analysis for the association of IL-12 levels with the risk of DR. Finally, the overall SMD of DR was 0.44 (95% CI (-0.81-1.70), *P* = 0.488) in IL-12 levels (Table 4). The publication bias was not significant.

#### 3.3.6. Analysis of IP-10

Two studies were included in the meta-analysis for the association of IP-10 level with the risk of DR. The pooled SMD for DR was 0.31 (95% CI (-0.16, 0.77), *P* = 0.193, Table 4) with substantial heterogeneity between studies (*I*^2^ = 20.5%, *P* = 0.262). The publication bias was not significant.

#### 3.3.7. Analysis of TNF-*α*

There were 4 studies which involved the aqueous humor levels of TNF-*α* and its risk for DR. Table 4 also shows that the pooled SMD of TNF-*α* for DR was 0.51 (95% CI (-0.04-1.06), *P* = 0.067) and without significant publication bias.

## 4. Discussion

To the best of our knowledge, the present meta-analysis evaluates the influence of the aqueous humor concentration of inflammatory cytokines (IL-6 among ten studies, IL-8 among ten studies, IL-10 among six studies, IL-12 among seven studies, TNF-*α* among six studies, MCP-1 among nine studies, and VEGF among thirteen studies) on the risk of DR or DME. In the current meta-analysis, we found that elevated levels of mediators or cytokines (VEGF, IL-6, and MCP-1) in the aqueous humor were strongly associated with DR, while the levels of VEGF, IL-6, IL-8, and MCP-1 were strongly associated with DME. Moreover, our meta-analysis presents strong points: the literature review was updated to comprise the latest evidence; a set of inclusion and exclusion criteria were applied for study selection; and outcomes were robust in the sensitivity or subgroup analysis.

In the current meta-analysis, there was a significant association between the aqueous humor VEGF levels and the risk for both DR and DME, and similar outcomes were also found in previous studies by Wu et al. [15] and Endo et al. [25]. VEGF is an endothelial cell mitogen, which can induce increases in vascular permeability and angiogenesis, enhance collateral vessel formation, and increase the permeability of the microvasculature [29]. Moreover, previous studies have pointed out that the VEGF level was significantly correlated with the severity of DR [30]. Recently, anti-VEGF drug intraventricular injection had been commonly used for the treatment of DR and DME [31, 32]. However, there are some complications associated with intravitreal injection, such as increased intraocular pressure (IOP) [33], endophthalmitis [34], and geographic atrophy [35]. Our results confirmed that VEGF levels in the aqueous humor have a significant correlation with both DR and DME. Therefore, the dosage form of VEGF may be changed to avoid the above complications and achieve the same therapeutic effect.

Another interesting finding of this meta-analysis is that levels of IL-6 in the aqueous humor have a significant relationship with DR and DME. It is well known that continuous proinflammatory responses and neovascularization are related to the occurrence and progression of DR. IL-6 is a proinflammatory cytokine and is a key factor in host defense against environmental stress such as inflammation, infection, and injury [36]. Moreover, IL-6 can also increase vascular permeability and angiogenesis by inducing VEGF expression [37]. Furthermore, Arjamaa et al. have documented that increased levels of IL-6 in the vitreous significantly correlated with the activity of neovascularization [38]. Furthermore, IL-6 could not only enhance inflammation responses of DR or DME but also contribute to neovascularization and promote the reaction with VEGF at the same time.

MCP-1 is also a proinflammatory cytokine, which can induce monocyte and macrophage infiltration into tissues and trigger their transmigration to the sites of inflammation produced by tissue hypoxia, infection, or macrophage injury [39–42]. Several studies reported that vitreous or aqueous levels of MCP-1 were higher in the eyes with DR compared with normal controls [7, 9]. They suggested that MCP-1 may play as a mediator in capillary occlusion in DR through the activation and adhesion of leukocytes and macrophages to the endothelium. Moreover, vitreous MCP-1 levels were found significantly correlated with the degree of proliferative membrane in the eyes with proliferative diabetic retinopathy (PDR), suggesting that MCP-1 may play an important role in the development of PDR [43]. In the current meta-analysis, due to the little information on the subtype of DR, we did not perform a meta-analysis on a different type of DR. Further studies are still needed to evaluate the association regarding cytokines in the aqueous humor and its risk for the severity of DR.

Moreover, previous studies have reported that the aqueous levels of MCP-1 were significantly higher in DME patients than in controls [8, 14]. Furthermore, the aqueous humor level of MCP-1 was significantly correlated with central macular thickness (CMT), suggesting that MCP-1 might play a role in the development of DME. MCP-1 is a potent eosinophil chemotactic cytokine and could recruit the monocytes to the sites of vascular injury [44]. Meanwhile, the monocytes and macrophages which accumulate on the wall of the blood vessel increase vascular permeability that potentiate DME [45]. In addition, the activity of MCP-1 seems to be closely related to VEGF because MCP-1 could mediate the gene expression of VEGF-A [46].

Indeed, IL-8 is the prototype of a CXC chemokine, which has been recognized as a potent chemoattractant and an activator of neutrophils and T lymphocytes [41, 47]. It is induced by hypoxia in vascular endothelial cells and plays an important role in promoting angiogenesis and tumor metastasis [41]. Once, some scholars reported that the levels of IL-8 in the vitreous were higher in the eyes with active PDR compared with quiescent PDR and they suggested that IL-8 might play an important part in the pathogenic process of PDR [48]. Elner et al. [49] also pointed out that the levels of IL-8 in the vitreous were significantly increased in active PDR. In our meta-analysis, we did not find any relationship between IL-8 and DR, maybe because we did not classify DR by severity. Moreover, researchers reported that IL-8 directly stimulates VEGF expression and the autocrine activation of VEGF receptor- (VEGFR-) 2 in vascular endothelial cells [19]. Kaneda et al. [50] reported that IL-8 appears to be involved in angiogenesis, endothelial cell binding and regeneration, endothelial wound healing, and vascular remodeling, presumably together with VEGF. These may be the reason why the aqueous humor levels of IL-8 increased significantly in the patients with DME.

Although some studies revealed that there are potential associations between the aqueous levels of IL-12, IP-10, or TNF-*α* and DR [15, 24], other outcomes regarding those associations are controversial [14, 20, 26, 27]. In our meta-analysis, there was no significant correlation between these cytokines and DR. Due to the lack of sufficient data, the association between cytokines (IL-12, IP-10, and TNF-*α*) and DME was not evaluated. Hence, further researches are needed for the evidence-based consequence.

The strengths of the current study include the comprehensive exploration of the evidence on the association between the cytokines in the aqueous humor and the risk of both DR and DME. However, there were some limitations in our study. First, there was a lack of studies available on the severity of DR for comparisons. Hence, our meta-analysis could only incorporate studies regarding any DR and controls. Second, we cannot obtain powerful outcomes adjusting potential confounders between the levels of cytokines and DR although we have done the subgroup analysis based on whether included studies had differences between case and control group or not. Last but not the least, there were insufficient studies for some cytokines such as IL-12, IP-10, and TNF-*α* to provide enough evidences to demonstrate the association between the biomarkers and risk for both DR and DME. Hence, large-sample, longitudinal, population-based studies are needed to validate our findings in the future.

## 5. Conclusion

In conclusion, we reviewed the literatures and conducted a current comprehensive meta-analysis to evaluate the association between the aqueous humor levels of VEGF, TNF-*α*, and inflammatory cytokines and risk for both DR and DME. Our findings indicated that lower mediator or cytokine levels in the aqueous humor may work best to attenuate DR and DME risk. This meta-analysis was the first comprehensive quantitative assessment of the aqueous humor levels of VEGF, IL-6, and MCP-1 on DR, which suggested that they may be used as biomarkers of the occurrence or development of DR, and reminded a new treatment besides intravitreal injection.

## Figures and Tables

**Table 1:** The characteristics of included studies about DR and control groups.

First author (year)	Country	Study design	DR definition	Measurements	DR			Non-DR			Cytokines
					Age (years)	M/F	Total	Age (years)	M/F	Total	
Shinoda (1999)	Japan	Case-control	ETDRS	ELISA	33-82	N.A	43	34-86	N.A	47	HGF and VEGF
Endo (2001)	Japan	Case-control	ETDRS	ELISA	74 ± 8	N.A	36	68.8 ± 13.5	N.A	102	VEGF and CML
Cheung (2012)	Singapore	Case-control	Dilated fundoscopic examination	Magnetic color bead-based multiplex assay	67.4 ± 10.7	18/9	27	65.9 ± 14.3	19/25	44	IL-1Ra, IL-1*β*, IL-2, IL-4, IL-5, IL-6, IL-7, IL-10, IL-12, IL-13, IL-17, G-CSF, GMCSF, IFN-*γ*, MCP-1 (CCL2), MIP-1*β* (CCL4), IL-8 (CXCL8), MIG (CXCL9), IP-10 (CXCL10), TNF-*α*, IFN-*α*, and VEGF
Gverović (2012)	Croatia	Case-control	ETDRS	ELISA	61-83	10/13	23	61-89	10/26	36	IL-12
Dong (2013)	China	Case-control	ETDRS	Multiplex bead immunoassay	N/A	N.A	N/A	69.3 ± 6.3	57/45	102	VEGF, IL-1*β*, IL-6, IL-8, MCP-1, IP-10, IL-10, and IL-12
Fulgêncio Cunha (2013)	Brazil	Case-control	N/A	ELISA	N/A	N.A	15	N.A	N.A	30	VEGF and IL-6
Kocabora (2015)	Turkey	Case-control	ETDRS	ELISA	65.5 ± 11.6	10/12	22	67.9 ± 9.2	22/23	45	TNF-*α* and CRP
Vujosevic (2016)	Italy	Case-control	N/A	RayBiotech technology	68.9 ± 11.4	6/5	11	74.7 ± 7.6	9/15	24	GM-CSF, IFN, IL, IP-10, MCP, M-CSF, MIP, RANTES, sTNF-R, and TNF
Chen (2017)	China	Case-control	DRDSS	Multiplex bead immunoassay	60.7 ± 7.6	54/47	101	60.0 ± 8.2	54/47	101	IL-1RA, IL-1*β*, IL-1*α*, IL-2, IL-4, IL-5, IL-6, IL-7, IL-8/CXCL8, IL-9, IL-10, IL-12 p70, IL-13, IL-15, IL-17A, IL-18, IL-21, IL-22, IL-23, IL-27, IL-31, TNF-*α*, TNF*β*/LTA, IFN-*γ*, and IFN-*α*
Wu (2017)	China	Case-control	Indirect ophthalmoscopy	Becton Dickinson CBA software	66-67	13/16	29	69.3	8/24	32	IL-1*β*, IL-2, IL-4, IL-5, IL-6, IL-10, IFN-*γ*, TNF, and VEGF
Khuu (2017)	Canada	Case-control	ETDRS	Luminex xMAP suspension array	67.5 ± 10.1	N.A	15	68.8 ± 6.35	N.A	17	FGF-1 & FGF-2, ANG-2, IL-8, leptin, stromal-derived factor-1, EGF, TGF-b1 and TGF-b2, G-CSF, PDGFs, VEGF, and ET-1
Houssen (2017)	Egypt	Case-control	FFA	ELISA	64.1 ± 4.5	N.A	40	39.2 ± 24.7	N.A	40	IL-27

N.A: not applicable; ETDRS: Early Treatment Diabetic Retinopathy Study; DRDSS: International Clinical Diabetic Retinopathy Disease Severity Scale; ELISA: enzyme-linked immunosorbent assay; HGF: human growth factor; VEGF: vascular endothelial growth factor; IL: interleukin; MCP: monocyte chemotactic protein; CML: carboxymethyl-lysine; G-CSF: granulocyte colony-stimulating factor; GMCSF: granulocyte macrophage colony-stimulating factor; IFN: interferon; MIP: macrophage inflammatory protein; MIG: monokine induced by IFN-*γ*; IP: interferon gamma-induced protein; M-CSF: macrophage colony-stimulating factor; RANTES: regulated and normal T cell expressed and secreted; sTNF-R: soluble tumor necrosis factor receptor; FGF: fibroblast growth factor; ANG: angiopoietins; TGF: transforming growth factor; EGF: epidermal growth factor; PDGF: platelet-derived growth factor; ET: erythropoietin, endothelin; M/F: male/female.

**Table 2:** Assessment of the quality of the included studies using the Newcastle-Ottawa scale (NOS).

First author	Quality evaluation	Case definition	Representativeness	Selection of controls	Definition of controls	Comparability	Ascertainment of exposure	Same method	Nonresponse rate
Shinoda	6	1	1	1	1	0	1	1	0
Endo	6	1	1	1	1	0	1	1	0
Funk	7	1	1	1	1	1	1	1	0
Oh	6	1	1	1	1	0	1	1	0
Lee	7	1	1	1	1	1	1	1	0
Cheung	6	1	1	1	1	0	1	1	0
Jonas	7	1	1	1	1	1	1	1	0
Gverović Antunica	6	1	1	1	1	0	1	1	0
Umazume	6	1	1	1	1	0	1	1	0
Dong	7	1	1	1	1	1	1	1	0
Fulgêncio Cunha	6	1	1	1	1	0	1	1	0
Kocabora	7	1	1	1	1	1	1	1	0
Vujosevic	7	1	1	1	1	1	1	1	0
Chen	7	1	1	1	1	1	1	1	0
Wu	7	1	1	1	1	1	1	1	0
Noma	6	1	1	1	1	0	1	1	0
Khuu	7	1	1	1	1	1	1	1	0
Houssen	7	1	1	1	1	1	1	1	0

**Table 3:** Subgroup analyses according to the location, diagnosis criteria of DR, and cytokine measurements.

	Number of studies	SMD (95% CI)	*P*	*I* ^2^	*P* ^∗^
Location					
Asia	6	1.40 (0.65, 2.14)	<0.001	93.40%	<0.001
South America	1	13.16 (10.31, 16.01)	<0.001	N.A	N.A
North America	1	0.23 (-0.46, 0.93)	0.513	N.A	N.A
Diagnosis criteria for DR					
ETDRS	5	1.32 (0.25, 2.40)	0.016	93.10%	<0.001
Dilated fundoscopic examination	1	2.01 (1.42, 2.59)	<0.001	N.A	N.A
International Clinical Diabetic Retinopathy Disease Severity Scale	1	0.31 (0.03, 0.59)	0.028	N.A	N.A
Indirect ophthalmoscopy	1	1.17 (0.62, 1.71)	<0.001	N.A	N.A
Cytokine measurements					
ELISA	3	4.26 (1.76, 6.75)	0.001	97.80%	<0.001
Luminex xMAP suspension array	1	0.23 (-0.46, 0.93)	0.513	N.A	N.A
Multiplex bead array assay	1	2.67 (1.79, 3.56)	<0.001	N.A	N.A
Magnetic color bead-based multiplex assay	1	2.01 (1.42, 2.59)	<0.001	N.A	N.A
Multiplex bead immunoassay	1	0.31 (0.03, 0.59)	0.028	N.A	N.A
Becton Dickinson CBA software	1	1.17 (0.62, 1.71)	<0.001	N.A	N.A

∗*P* value for heterogeneity. SMD: standardized mean difference; ETDRS: Early Treatment Diabetic Retinopathy Study; N.A: not applicable; ELISA: enzyme-linked immunosorbent assay.

**Table 4:** The associations between IL-8 with DR and DME and IL-12, IP10, and TNF-*α* with DR.

	Number of studies	SMD (95% CI)	*P*	*I* ^2^	*P* ^∗^
IL-8 & DR					
Total	3	0.38 (-0.05, 0.81)	0.078	60.80%	0.087
IL-8 & DME	5	1.68 (0.97, 2.40)	<0.001	81.00%	<0.001
Subgroup					
*Countries*					
Asian	4	1.86 (0.95, 2.77)	<0.001	83.90%	<0.001
European	1	1.08 (0.45, 1.71)	0.001	N.A	N.A
*DR diagnosis criteria*					
ETDRS	3	2.05 (0.61, 3.49)	0.005	88.90%	<0.001
Diabetic Retinopathy Disease Severity Scale	1	1.49 (1.04, 1.93)	<0.001	N.A	N.A
*Cytokine measurements*					
Luminex xMAP suspension array	3	1.84 (0.42, 3.25)	0.011	90.50%	<0.001
BD Cytometric Bead Array	1	1.61 (0.68, 2.54)	0.001	N.A	N.A
Multiplex bead immunoassay	1	1.49 (1.04, 1.93)	<0.001	N.A	N.A
*Remove each study for sensitivity analysis*					
Lee et al., 2012	4	1.87 (1.0, 2.74)	<0.001	83.90%	<0.001
Jonas et al., 2012	4	1.86 (0.95, 2.77)	<0.001	83.90%	<0.001
Umazume et al., 2013	4	1.71 (0.85, 2.58)	<0.001	85.70%	<0.001
Chen et al., 2017	4	1.77 (0.72, 2.82)	0.001	85.70%	<0.001
Noma et al., 2017	4	1.32 (1.01, 1.62)	<0.001	0%	0.525
IL-12 & DR					
Total	3	0.44 (-081, 1.70)	0.488	94.20%	<0.001
IP-10 & DR					
Total	2	0.31 (-0.16, 0.77)	0.017	20.50%	0.262
TNF-*α* & DR					
Total	4	0.51 (-0.04, 1.06)	0.067	80.20%	0.002

^∗^ *P* value for heterogeneity.

## Data Availability

The data used to support the findings of this study are included within the article.

## Supplementary Materials

Supplementary Materials The basic clinical information and characteristics of each included study about diabetic macular edema (DME) on the system.
