# Supplementary material for: Aqueous Humor Mediator and Cytokine Aberrations in Diabetic Retinopathy and Diabetic Macular Edema: A Systematic Review and Meta-Analysis
Source: Dis Markers. 2019 Nov 23;2019:6928524. doi: 10.1155/2019/6928524 (PMC6906842; doi:10.1155/2019/6928524)
Supplement: Supplementary Materials — The basic clinical information and characteristics of each included study about diabetic macular edema (DME) on the system. [file 6928524.f1.doc]

**Table S1. The basic clinical informations and characteristics of each study about DME.**

| First author  (year) | Country | Study design | DR definition | Measurements | DME | |  | Non-DME | | Cytokines |
| --- | --- | --- | --- | --- | --- | --- | --- | --- | --- | --- |
|  |  |  |  |  | Age (years) | M/F |  | Age (years) | M/F |  |
| Funk M  (2010) | Austria | Case-control | ICDMEDSS | Luminex xMAP suspension array | 67.9±10.1 | N.A |  | 69.9±3.5 | N.A | IL-6, IL-8, MCP-1, and VEGF |
| Lee WJ  (2012) | Korea | Case-control | CSMT 250 mm or greater on OCT | Luminex xMAP suspension array | 54.8±12.3 | 9/9 |  | 66.9±7.3 | 4/12 | IL-2, IL-5, IL-6, IL-8, IL-12p70, IL-13, MCP-1, MIP-1α, PDGF-AA, TGF-α, IFN-γ, EGF, FGF2, and VEGF |
| Jonas JB  (2012) | Germany | Case-control | ETDRS | Luminex xMAP suspension array | 70.9±9.3 | 15/8 |  | 74.8±10.8 | 12/10 | TGF-a, TGF-b, EGF, FGF-beta, HGF, IFN-a, IFN-b, IFN-g, IL-1a2, IL-1b, IL-2, IL-3, IL-4, IL-5, IL-6, IL-8, IL-10, IL-12p40, IL-12p70, IP10, ICAM-1, MCP-1, MCP-3, MMIF, MIG, MMP-1, MMP-9, PAI1, PlGF, PDGF-BB, TRAIL, VCAM, and VEGF |
| Umazume K  (2013) | Janpan | Case-control | ETDRS | BD Cytometric Bead Array Flex Set System and BD Human Soluble Protein Master Buffer Kit | 68.2±7.8 | 6/8 |  | 65.3±8.7 | 9/15 | sCD14, IL-8, IP-10 Mig, MCP-1, and VEGF |
| Kocabora MS  (2015) | Turkey | Case-control | ETDRS | ELISA | N.A | 10/10 |  | N.A | 10/10 | TNF-a, and CRP |
| Chen H  (2017) | China | Case-control | DRDSS | multiplex bead immunoassay | 55 | N.A |  | 46 | N.A | IL-1RA, IL-1β, IL-1α, IL-2, IL-4, IL-5, IL-6, IL-7, IL-8/CXCL8, IL-9, IL-10, IL-12 p70, IL-13, IL-15, IL-17A, IL-18, IL-21, IL-22, IL-23, IL-27, IL-31, TNF-α, TNFβ/LTA, IFN-γ, and IFN-α |
| Noma H  (2017) | Janpan | Case-control | ETDRS | Luminex xMAP suspension array | 64.3±10.3 | 24/11 |  | 66.7±4.7 | 8/5 | sVEGFR-1, sVEGFR-2, VEGF, PlGF, sICAM-1, MCP-1, PDGF-AA, IL-6, IL-8, IL-12p70, IL-13, and IP-10 |

N.A: Not Applicable; ICDMEDSS: International Clinical Diabetic Macular Edema Disease Severity Scale; ETDRS: Early Treatment Diabetic Retinopathy Study; DRDSS: Diabetic Retinopathy Disease Severity Scale; ELISA: enzyme linked immunosorbent assay; IL: Interleukin; MCP: Monocyte chemotactic protein; IP: Interferon gamma-induced protein; VEGF: vascular endothelial growth factor; MIP: macrophage inflammatory protein; PDGF: platelet-derived growth factor; TGF: transforming growth factor; IFN: interferon; FGF: fibroblast growth factor; EGF: epidermal growth factor; HGF: human growth factor; ICAM: intercellular adhesion molecule; MMP: matrix metalloproteinase; MMIF: macrophage migration inhibitory factor; MIG: monokine induced by IFN-γ; PAI: plasminogen activator inhibitor; PlGF: placenta growth factor; TRAIL: tumor necrosis factor alpha–related apoptosis inducing ligand; PDGF BB: platelet-derived growth factor BB; VCAM: vascular cell adhesion molecule; sCD14: soluble CD14; CRP: C-reactive protein; sVEGFR: soluble vascular endothelial growth factor receptor; PlGF: placental growth factor; M/F: male/female.
